# Kinematic Analysis of the Postural Demands in Professional Soccer Match Play Using Inertial Measurement Units

**DOI:** 10.3390/s20215971

**Published:** 2020-10-22

**Authors:** José M. Oliva-Lozano, Elisa F. Maraver, Víctor Fortes, José M. Muyor

**Affiliations:** 1 Health Research Centre, University of Almería, 04120 Almería, Spain; josemuyor@ual.es; 2 Faculty of Computer Science, Multimedia and Telecommunications, Universitat Oberta de Catalunya, 08018 Barcelona, Spain; elisafm@uoc.edu; 3 Unión Deportiva Almería, 04007 Almería, Spain; vifosa@gmail.com; 4 Laboratory of Kinesiology, Biomechanics and Ergonomics (KIBIOMER Lab.), Research Central Services, University of Almería, 04120 Almería, Spain

**Keywords:** football demands, running posture, game analysis, load management, team sports, tracking systems

## Abstract

The development of wearable sensors has allowed the analysis of trunk kinematics in match play, which is necessary for a better understanding of the postural demands of the players. The aims of this study were to analyze the postural demands of professional soccer players by playing position. A longitudinal study for 13 consecutive microcycles, which included one match per microcycle, was conducted. Wearable sensors with inertial measurement units were used to collect the percentage (%) of playing time spent and G-forces experienced in different trunk inclinations and the inclination required for different speeds thresholds. The inclination zone had a significant effect on the time percentage spent on each zone (*p* < 0.001, partial eta-squared (*ηp*^2^ = 0.85) and the G-forces experienced by the players (*p* < 0.001, *ηp*^2^ = 0.24). Additionally, a significant effect of the speed variable on the trunk inclination zones was found, since trunk flexion increased with greater speeds (*p* < 0.001; *ηp*^2^ = 0.73), except for midfielders. The players spent most of the time in trunk flexion between 20° and 40°; the greatest G-forces were observed in trunk extension zones between 0° and 30°, and a linear relationship between trunk inclination and speed was found. This study presents a new approach for the analysis of players’ performance. Given the large volumes of trunk flexion and the interaction of playing position, coaches are recommended to incorporate position-specific training drills aimed to properly prepare the players for the perception-action demands (i.e., visual exploration and decision-making) of the match, as well as trunk strength exercises and other compensatory strategies before and after the match.

## 1. Introduction

Soccer is a team sport that is played in a dynamic environment, with considerable demands on the perceptual-motor skills of the players [1,2,3]. Then, as in other team sports in which the ball, teammates, referees, or opposition players are continually in motion, it is suggested that the understanding of the postural demands met by the players when performing sports-specific skills would provide coaches and performance analysts with meaningful information about their performance in perception and action [4]. For example, the downward orientation of the head and the trunk may restrict the ability to perform in the field of regard [4,5]. However, soccer players usually play the ball with the feet, which may increase the trunk flexion and move the field of regard down [4]. Additionally, the increase in trunk flexion is a natural movement given the increase in speed [6], which suggests that the analysis of the trunk inclination required for different speeds thresholds is necessary.

In addition, the trunk kinematics have a significant effect on knee and hip energetics in running [7], hamstring injury [8], patellofemoral joint stress [9], and low back pain in professional soccer players [10]. For instance, a previous study found that upright trunk posture in running was associated with greater patellofemoral joint stress than running with forward trunk flexion [9]. In this regard, another study concluded that running with an upright trunk posture increased knee extensors’ energy generation and absorption, while incorporating ~10° trunk flexion decreased such energy generation and absorption [7].

Moreover, soccer is a sport that involves different high-intensity actions, such as accelerations, decelerations, sprints, changes of directions, jumps, collisions, or landings [11,12,13,14]. In this context, the ankles, knees, and hips chained to the spine play a crucial role as shock absorbers, since the magnitude and total of shocks on the musculoskeletal system lead to chronic injury risks in recreational runners [15,16]. For instance, the trunk acceleration magnitude (i.e., G-force) is highly correlated to the forces lending to the mechanics of injury [16]. These accelerations, which are measured by inertial measurement units that register triaxial movements (*x*, *y*, and *z*), are considered as an important external workload indicator [17,18]. In consequence, the understanding of the G-forces placed on the trunk when performing activities involving running actions is considered necessary in order to maximize running economy and injury prevention [17].

However, there are limited data available to date concerning the postural demands of professional soccer players in match play [17]. A recent investigation, which was conducted on soccer players [17], suggested that practitioners should consider this information for the design of training drills, given the postural demands observed during match play. In addition, this investigation concluded that strength and conditioning coaches should also consider the impact that contextual variables such as playing position may have on the trunk inclination and G-forces experienced by the players [17]. Nevertheless, more studies on the postural demands of professional soccer players during match play are necessary, because it is the only study published to date [17]. Perhaps one of the reasons for the lack of research on this topic is explained by the methodological difficulties associated with the instruments used for measuring trunk kinematics [19]. Although the gold standard instruments for this aim are the motion capture systems, these are limited to laboratory settings [19,20]. In recent years, wearable inertial measurement units have been considered as alternative instruments for motion capture, since these may give insight into how soccer players use their trunk to perform the sport-specific skills in match play [4,21].

Therefore, the aims of this study were to: (1) analyze the percentage (%) of playing time that soccer players spend in different trunk inclinations in match play; (2) analyze the G-forces that soccer players experience in different trunk inclinations; (3) analyze the trunk inclination required for different speeds thresholds; and (4) analyze the effect of playing position on the time percentage that soccer players spend in different trunk inclinations, G-forces that the players experience in different trunk inclinations, and trunk inclination required for different speeds thresholds. Based on the results observed from a recent study on professional soccer players [17], it is hypothesized that the players may spend most of the time in trunk flexion. Then, the greatest G-forces may be observed in similar trunk flexion ranges, and a linear relationship between trunk inclination and speed should be found.

## 2. Materials and Methods

### 2.1. Study Design

A longitudinal study for 13 consecutive microcycles, which included one match per microcycle, was conducted in LaLiga 123. This study was carried out between 17 March 2019 (first match) and 6 June 2019 (last match). Wearable sensors with inertial measurement units were used to obtain time-, acceleration-, and velocity-based variables considering different trunk inclination angles. The study was designed according to the Ethical Standards in Sports and Exercise Science Research [22], and the club authorized the data collection during the competitive season. In addition, the institutional bioethics committee’s approval was obtained.

### 2.2. Participants

A total of 15 professional soccer players (27.14 ± 3.94 years; 1.82 ± 0.06 cm; 75.57 ± 5.64 kg) participated in the study. The players were categorized into different playing positions: central defenders (CD), full-backs (FB), forwards (FW), midfielders (MF), and wide-midfielders (WMF). Only players who completed the total duration of the match were included in the analysis. However, goalkeepers were not considered for the study, given the different nature of their activity profile [23].

### 2.3. Procedures

**Figure 1:**
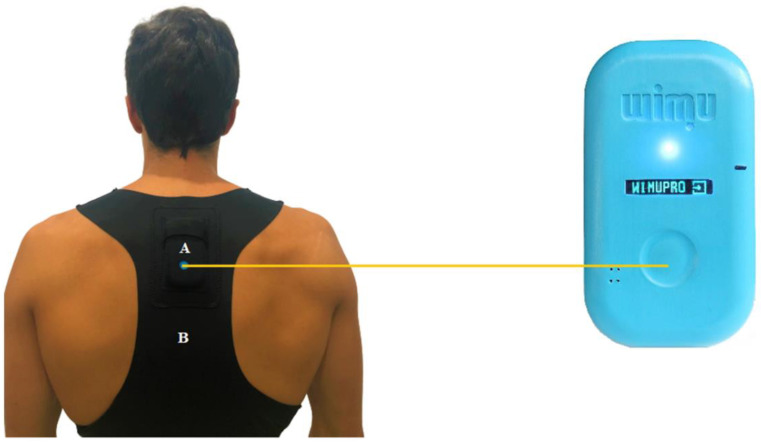
Tracking system (**A**) placed in the back pocket of a chest vest (**B**).

The data were collected by WIMU Pro devices (RealTrack Systems, Almería, Spain) in match play (Figure 1). These are wireless inertial measurement units composed of four triaxial accelerometers, three triaxial gyroscopes, one triaxial magnetometer, and one barometer. In addition, these devices are global positioning systems (GPS). Based on previous studies that used the same tracking devices [11,24], these were calibrated before the start of the match following the manufacturer’s instructions (RealTrack Systems, Almería, Spain). The data collected by the device was visualized on SPro (RealTrack Systems, Almería, Spain) at the end of each match, and the raw data from the “ATTITUDE EULER Z”, “ACELT”, and “GPS Speed” channels was downloaded for the analysis.

Since each player wore a WIMU Pro device, which was vertically placed in the back pocket of a chest vest, the “ATTITUDE EULER Z” represented the orientation of the device (in degrees) for movements within the *z*-axis (i.e., trunk flexion and extension movements) [19]. This device has been considered as a reliable instrument for measuring the inclination for movements within the *z*-axis (mean bias: ~1.2°; intraclass correlation coefficients >0.97; coefficient of variation <7%) [19]. Then, these data, which were collected at 100 Hz, were used to calculate the trunk inclination zones based on a recent protocol [4]. Considering that the upright position represented the 0° position and the “ATTITUDE EULER Z” reported −90° for this position, the authors applied a formula (i.e., value from “ATTITUDE EULER Z plus 90”) in order to obtain the upright position at 0°. Therefore, the trunk flexion zones were: 0°–10°, 10°–20°, 20°–30°, 30°–40°, 40°–50°, 50°–60°, 60°–70°, 70°–80°, 80°–90°, and >90°, while the trunk extension zones were: 0° to −10°, −10° to −20°, −20° to −30°, −30° to −40°, and <−40°. In addition, the time that soccer players spent in different trunk inclinations were calculated as the time percentage (e.g., total of values in 10°–20° zone/total duration ×100), since the matches had different durations (i.e., 90 min plus match-related stoppage time).

**Figure 2:**
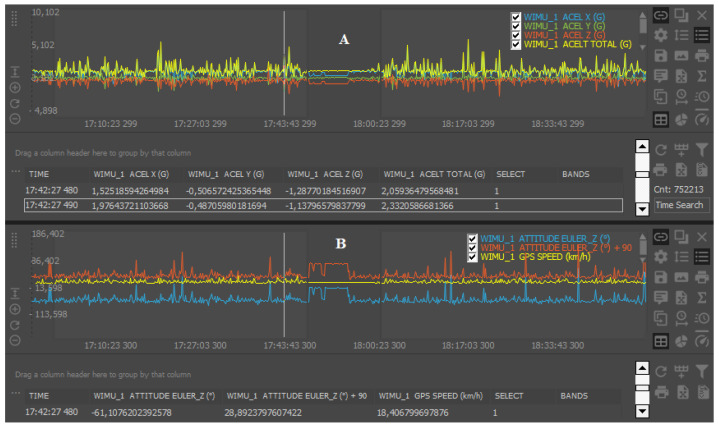
Raw data collected by the tracking system from the “ACELT” channel (**A**) and the “ATTITUDE EULER Z plus 90” (i.e., upright position) synchronized with global positioning system “GPS Speed” (**B**).

The “ACELT” is defined as the resultant vector of the G-forces registered by the triaxial accelerometers (*x*, *y*, and *z*), which measures the combination of gravity and changes in vertical and horizontal movements of the device (“ACELT” formula = $\sqrt{x^{2}+y^{2}+z^{2}}$) [25] (Figure 2A). The accelerometers from WIMU Pro (RealTrack Systems, Almería, Spain) were tested and considered as good instruments, given the accuracy to calculate accelerometry-based variables (mean bias: ~0.01 G; coefficient of variation <0.6%) [25].

Finally, the “GPS Speed” data were collected at 10 Hz. The speed data collected by WIMU Pro was considered as valid (bias: 1.2–1.3 km/h) and reliable (intraclass correlation coefficients: >0.93) [26]. The GPS data collected were synchronized with the “EULER Z” data in order to calculate the players’ average trunk inclinations required for different speed thresholds (0–7, 7–14, 14–21, and >21 km/h) (Figure 2B).

### 2.4. Statistical Analysis

The descriptive statistics were obtained for the time percentage that soccer players spent in each zone of trunk inclination, the G-forces that the players experienced in each zone of trunk inclination, and the trunk inclination required for each speed threshold. Shapiro-Wilk test was used to test the normality of the data, and Levene’s test was performed to assess the equality of variances. The sphericity was obtained through Mauchly’s test (*p* < 0.05 in all variables). A linear model with mixed-design analysis of variance for repeated measures was performed. Playing position was considered a between-subject variable for this analysis. The comparisons between the time percentage that the soccer players spent in each zone of trunk inclination, the G-forces that the players experienced in each zone of trunk inclination, and the trunk inclination required for each speed threshold were obtained through Bonferroni post hoc. Additionally, the effect sizes were reported through partial eta-squared (*ηp*^2^). The statistical analysis was run on SPSS Statistics for Windows (IBM Corp., Armonk, NY, USA), with the level of significance set at *p* ≤ 0.05.

## 3. Results

Table 1 shows the descriptive statistics with the time percentage that the soccer players from each playing position spent on different inclination zones during match play, as well as the differences between zones. The inclination zone variable had a significant effect on the time percentage spent on each zone (*F*_(1.18, 154.06)_ = 510.1; *p* < 0.001; *ηp*^2^ = 0.85). Specifically, the soccer players spent most of the time in trunk flexion between 20° and 30° (WMF: ~50%, CD: ~46%, MF: ~45%, FB: ~42%, and FW: ~36%), followed by the zone between 30° and 40° (FW: ~37%, CD: ~36%, FB: ~26%, MF: ~24%, and WMF: 21%). In addition, a significant interaction between playing position and the time percentage spent in different trunk inclination zones was observed (*F*_(7.37, 154.06)_ = 5.74; *p* < 0.001; *ηp*^2^ = 0.21).

Table 2 shows the descriptive statistics with the G-forces that the soccer players from each playing position experienced in different inclination zones during match play, as well as the differences between zones. The inclination zone also had a significant effect on the G-forces that the soccer players experienced in each zone (*F*_(2.85, 247.96)_ = 26.78; *p* < 0.001; *ηp*^2^ = 0.24). Specifically, the greatest G-forces were observed in trunk extension zones for CD (~2.3 G between 10° and 20° trunk extension), MF (~2.3 G between 0° and 10° trunk extension), WMF (~2.2 G between 10° and 20° trunk extension), FW (~1.9 G between 20° and 30° trunk extension), and FB (~1.8 G between 0° and 10° trunk extension). However, the interaction between playing position and the G-forces experienced in the trunk inclination zones was not significant (*F*_(11.4, 87)_ = 0.93; *p* = 0.52; *ηp*^2^ = 0.04).

**Figure 3:**
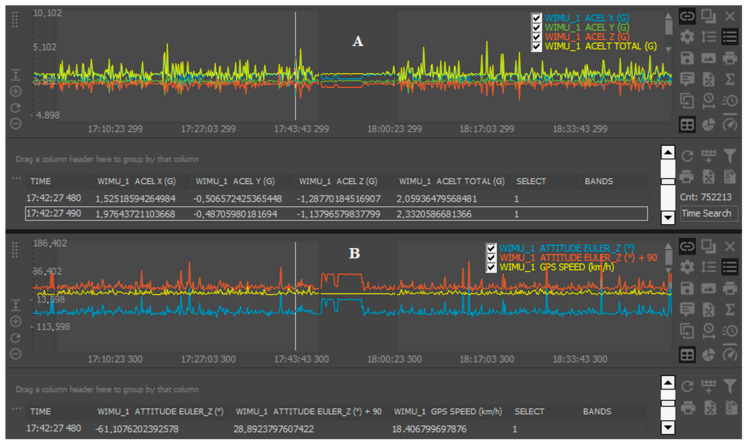
Trunk inclination (in degrees) required for different speed thresholds and playing positions. Central defenders (CD), full-backs (FB), forwards (FW), midfielders (MF), and wide-midfielders (WMF). a Statistical difference to 0–7 km/h (*p* < 0.05), b statistical difference to 7–14 km/h (*p* < 0.05), c statistical difference to 14–21 km/h (*p* < 0.05), and d statistical difference to ≥21 km/h (*p* < 0.05).

Regarding the analysis of the trunk inclination required for different speeds thresholds (Figure 3), a significant effect of the speed variable was found, since trunk flexion increased with greater speeds (*F*_(1.83, 162.85)_ = 239.52; *p* < 0.001; *ηp*^2^ = 0.73). However, the greatest trunk inclination for MF (i.e., 31.84° ± 0.85°) was found between 7 and 14 km/h. In addition, a significant interaction between playing position and the trunk inclination required for the speed zones was observed (*F*_(7.31, 89)_ = 18.55; *p* < 0.001; *ηp*^2^ = 0.45).

## 4. Discussion

The aims of this study were to analyze the time percentage that soccer players spent in different trunk inclinations in match play, analyze the G-forces experienced in different trunk inclinations, and analyze the trunk inclination required for different speeds thresholds, while considering the effects of playing positions. This study is one of the first steps in the analysis of the postural demands of professional soccer players. The main findings were that these players spent most of the time in trunk flexion between 20° and 40°, the greatest G-forces were observed in trunk extension zones between 0° and 30°, and a linear relationship between trunk inclination and speed in all playing positions was observed, except for MF. Therefore, the results do not confirm the hypothesis that the greatest G-forces may be observed in trunk flexion zones.

The results showed that the soccer players spent most of the time in trunk flexion between 20° and 40°. A recent investigation on field hockey players observed that these were half of a match in two trunk flexion zones (30°–40°: ~26% of total time and 40°–50°: ~26% of total time) [4]. This trunk inclination allowed the players to perform the sport-specific movements such as running with the ball or passing and controlling the ball, which may move the field of regard down [4,5]. In addition, the results showed that the playing position should be also considered when analyzing the postural demands. WMF was the position with the greatest time percentage (~50%) between 20° and 30°, while FW showed the lowest time percentage (~36%) in match play. Although these differences may be due to the positional demands (i.e., WMF tend to run with the ball for longer times than FW) [27], these results suggest that these postural demands (e.g., time percentage that the players spend in trunk flexion) need to be considered when designing specific training drills [28]. This becomes even more important when players report low back pain, which is associated with altered lumbopelvic control [10], because the players may tend to adopt trunk-flexed postures [29,30]. Hence, trunk strength exercises and lumbopelvic control exercises may be also beneficial for the players [10,28].

When it comes to the G-forces that the soccer players experienced in different inclination zones during match play, a novel finding of the study was that the greatest G-forces were observed in trunk extension zones for all playing positions. Given the short period of time that the players spent in trunk extension zones during the matches (below 2% of the total time), these results might be explained by the fact that the players suffer from very specific collisions, falls, or jumps in these brief periods [11,31]. Nonetheless, the results from this study showed that the average G-forces of soccer players in trunk flexion (~1.32 G) during match play are consistent with previous research on running kinematics, which examined the average G-forces experienced by the athletes (1.21–1.38 G) [16]. However, contrary to the findings on the previous aim, which examined the time percentage that the players spent in each trunk inclination zone, no significant effect of playing position on the G-forces experienced in the trunk inclination zones was found. In this regard, future studies are needed in order to understand if the postural demands are dependent on the playing position or the player itself.

Finally, the results showed that the trunk flexion increased with greater speeds in all playing positions, except for MF. Although the trunk inclination is a variable that has received little attention in the literature, it is to highlight that the increase in trunk flexion is a natural movement given the increase in speed [6]. The progression to faster running speeds is associated with increases in electromyographic activity, especially in the multifidus muscle, and kinematic changes [32]. However, MF showed different results, because the greatest trunk inclination was observed between 7 and 14 km/h, which implies that postural demands of the MF players differ from the rest of the playing positions. Considering the importance of the tactical connection between MF and the rest of the playing positions in addition to their role as game organizers (e.g., passing the ball and linking the sectors of the team) [33], these results may be explained by the fact that this position requires an upright posture at greater speeds in order to perform in their field of regard [4,5]. From a practical perspective, this is one of the reasons why previous researchers suggested that it is important to understand the postural demands of the players during the sport-specific skills [4,21,34]. For example, if a player tends to increase trunk flexion when sprinting, this may lead to the downward orientation of the head and restrict the ability to perform in the field of regard [4,5]. In consequence, training drills focused on visual exploration and decision-making are necessary in order to have a successful performance in the field of regard.

It is plausible that some limitations of the study may influence the interpretations of these results. For example, data was collected from only one professional soccer team. This study was focused on the trunk kinematics, but an analysis of the kinematics of the ankles, knees, or hips was not conducted. Only players who completed the total duration of the match were included in the analysis as homogenized criteria. In addition, not all the playing positions could be analyzed, since goalkeepers were not included. Therefore, future studies should consider these limitations in addition to other variables of interest, such as ball possession or player’s fatigue, which may have a significant relationship with the postural demands of soccer players [17]. For instance, the trunk inclination and G-forces experienced by professional soccer players in match play are usually reduced during the second half of the match, which suggests that fatigue may lead to changes in the kinematics of the players [17].

In addition, the data were collected by inertial measurement units and GPS technology, which have advantages and disadvantages from a practical standpoint. For example, this technology allows the collection of external load (e.g., kinematic parameters such as trunk inclination or speed) and internal load variables (e.g., heart rate) [35,36]. Although these instruments are valid, reliable, and suitable for measuring the inclination in the *z*-axis [19], acceleration (G-forces) [25], and position-related variables [26], the gold standard technology for motion capture are optical tracking systems [20]. Optical tracking systems are limited to laboratory settings, and thus, wearable sensors have been considered as an alternative method for motion capture [20]. However, previous investigations suggested that the inertial measurement units should include methods to compensate for the drift error and improve the accuracy of the data [37,38,39]. Specifically, the multi-sensor fusion and the placement of multiple sensors on different segments of the body are frequent methods that may increase the accuracy of the data collected [37,38,39,40,41]. Nonetheless, this last method is useful in professional soccer, because wearing multiple sensors on the body may be dangerous and uncomfortable for the player [17,42].

## 5. Conclusions

This study showed how the data collected by inertial measurement units may be used for the analysis of the postural demands of professional soccer players. Since the results showed that match play led to significant postural demands, coaches are recommended to incorporate training drills that consider the match demands. For example, the volume of trunk flexion observed implies that soccer players may unconsciously move the field of regard down, and position-specific training drills at different speeds are necessary in order to properly prepare the players for the perception-action demands (i.e., visual exploration and decision-making) of the match. In addition, it is suggested that trunk strength exercises are designed with special focus on flexion positions, as well as other compensatory exercises for trunk extension muscles, which balance trunk flexors and trunk extensors. Indeed, the trunk strength exercises might add perturbations so as to stimulate trunk accelerations between 2 and 3 G in both flexion and extension.

## Figures and Tables

**Table 1:** Time percentage (%) spent on each trunk flexion and extension zone (mean ± standard deviation).

	Inclination Zones (°)	Central Defenders	Full-Backs	Forwards	Midfielders	Wide-Midfielders
Trunk flexion	0–10	0.17 ± 0.18 c, d, e, f, j	1.05 ± 1.25 b, c, d, e, h, i, k, l, m, n, o	0.47 ± 0.80 c, d, e, f, g, j	0.58 ± 0.60 b, c, d, e, f	0.95 ± 0.50 b, c, d, e, f, k, l, m, n, o
	10–20	3.29 ± 3.43 c, d	18.95 ± 18.10 a, c, f, g, h, i, j, k, l, m, n, o	7.08 ± 8.79 c, d	17.31 ± 13.58 a, c, f, g, h, i, j, k, l, m, n, o	13.41 ± 4.09 a, c, f, g, h, i, j, k, l, m, n, o
	20–30	46.09 ± 11.52 a, b, e, f, g, h, i, j, k, l, m, n, o	42.16 ± 13.31 a, b, e, f, g, h, i, j, k, l, m, n, o	36.16 ± 13.72 a, b, e, f, g, h, i, j, k, l, m, n, o	44.62 ± 12.43 a, b, d, e, f, g, h, i, j, k, l, m, n, o	50.20 ± 4.21 a, b, d, e, f, g, h, i, j, k, l, m, n, o
	30–40	35.47 ± 10.40 a, b, e, f, g, h, i, j, k, l, m, n, o	25.59 ± 13.91 a, e, f, g, h, i, j, k, l, m, n, o	36.75 ± 14.36 a, b, e, f, g, h, i, j, k, l, m, n, o	24.23 ± 14.35 a, c, e, f, g, h, i, j, k, l, m, n, o	20.71 ± 4.26 a, c, e, f, g, h, i, j, k, l, m, n, o
	40–50	9.47 ± 3.12 a, c, d, f, g, h, i, j, k, l, m, n, o	7.79 ± 5.39 a, c, d, f, g, h, i, j, k, l, m, n, o	10.67 ± 3.89 a, c, d, f, g, h, i, j, k, l, m, n, o	8.59 ± 4.65 a, c, d, f, g, h, i, j, k, l, m, n, o	8.15 ± 1.74 a, c, d, f, g, h, i, j, k, l, m, n, o
	50–60	3.05 ± 1.36 a, c, d, e, g, h, i, j, k, l, m, n, o	2.40 ± 1.84 b, c, d, e, g, h, i, j, k, l, m, n, o	3.70 ± 1.04 a, c, d, e, g, h, i, j, k, l, m, n, o	2.76 ± 1.39 a, b, c, d, e, g, h, i, j, k, l, m, n, o	3.08 ± 0.63 a, b, c, d, e, g, h, i, j, k, l, m, n, o
	60–70	0.84 ± 0.43 c, d, e, f, g, h, i, k, l, m, n	0.80 ± 0.65 b, c, d, e, f, l, m, n	1.92 ± 2.23 a, c, d, e, f, h, i, k, l, m, n, o	0.79 ± 0.31 b, c, d, e, f	1.08 ± 0.26 b, c, d, e, f, k, l, m, n, o
	70–80	0.28 ± 0.14 c, d, e, f, g, j, k, l, m, n	0.31 ± 0.23 a, b, c, d, e, f, g, k, l, m, n	0.64 ± 0.48 c, d, e, f, g, j, k, l, m, n	0.29 ± 0.18 b, c, d, e, f, k, l, m, n, o	0.46 ± 0.19 b, c, d, e, f, j, k, l, m, n, o
	80–90	0.16 ± 0.07 c, d, e, f, g, j	0.16 ± 0.09 a, b, c, d, e, f, j	0.47 ± 0.56 c, d, e, f, g, j, k, l, m, n	0.18 ± 0.18 b, c, d, e, f	0.41 ± 0.18 b, c, d, e, f, j, k, l, m, n, o
	>90	1.01 ± 0.66 a, c, d, e, f, h, i, k, l, m, n, o	0.68 ± 0.28 b, c, d, e, f, i, k, l, m, n, o	1.85 ± 0.90 a, c, d, e, f, h, i, k, l, m, n, o	0.60 ± 0.69 b, c, d, e, f, l, m, n	1.45 ± 0.70 b, c, d, e, f, h, i, k, l, m, n, o
Trunk extension	0–10	0.03 ± 0.03 c, d, e, f, g, h, j	0.06 ± 0.06 a, b, c, d, e, f, h, j, l, m, n	0.04 ± 0.01 c, d, e, f, g, h, i, j	0.03 ± 0.02 b, c, d, e, f, h, m, n	0.06 ± 0.03 a, b, c, d, e, f, g, h, i, j, l, m, n
	10–20	0.01 ± 0.00 c, d, e, f, g, h, j	0.01 ± 0.01 a, b, c, d, e, f, g, h, i, j, k, m, n	0.02 ± 0.01 c, d, e, f, g, h, i, j, m	0.01 ± 0.01 b, c, d, e, f, h, j, m, n	0.01 ± 0.01 a, b, c, d, e, f, g, h, i, j, k, m, n
	20–30	0.00 ± 0.00 c, d, e, f, g, h, j	0.00 ± 0.00 a, b, c, d, e, f, g, h, j, k, l	0.01 ± 0.01 c, d, e, f, g, h, i, j, l	0.00 ± 0.00 b, c, d, e, f, h, j, k, l	0.01 ± 0.01 a, b, c, d, e, f, g, h, i, j, k, l
	30–40	0.00 ± 0.00 c, d, e, f, g, h, j	0.00 ± 0.01 a, b, c, d, e, f, g, h, j, k, l	0.02 ± 0.01 c, d, e, f, g, h, i, j	0.00 ± 0.00 b, c, d, e, f, h, j, k, l	0.00 ± 0.00 a, b, c, d, e, f, g, h, i, j, k, l
	>40	0.15 ± 0.60 c, d, e, f, j	0.05 ± 0.21 a, b, c, d, e, f, j	0.20 ± 0.36 c, d, e, f, g, j	0.01 ± 0.03 b, c, d, e, f	0.02 ± 0.04 a, b, c, d, e, f, g, h, i, j

Note: a Statistical difference to 0°–10° (flexion), b statistical difference to 10°–20° (flexion), c statistical difference to 20°–30° (flexion), d statistical difference to 30°–40° (flexion), e statistical difference to 40°–50° (flexion), f statistical difference to 50°–60° (flexion), g statistical difference to 60°–70° (flexion), h statistical difference to 70°–80° (flexion), i statistical difference to 80°–90° (flexion), j statistical difference to >90° (flexion), k statistical difference to 0°–10° (extension), l statistical difference to 10°–20° (extension), m statistical difference to 20°–30° (extension), n statistical difference to 30°–40° (extension), and o statistical difference to >40° (extension).

**Table 2:** “ACELT” (G) for each trunk flexion and extension zone (mean ± standard deviation).

	Inclination Zones (°)	Central Defenders	Full-Backs	Forwards	Midfielders	Wide-Midfielders
Trunk flexion	0–10	2.16 ± 0.45 b, c, d, e, f, g, h, i, j	1.49 ± 0.26 b, c,	1.83 ± 0.32 b, c, d, e, h, i, j	2.12 ± 0.69 b, c, d, e, f, g, h, i, j	1.56 ± 0.15 b, c, i, j, k
	10–20	1.48 ± 0.22 a, c, d, e, h, i, j, k, l	1.20 ± 0.12 a, f, g, k	1.30 ± 0.14 a, c, g, k	1.27 ± 0.22 a, c, l, k	1.19 ± 0.04 a, f, g, k, l
	20–30	1.09 ± 0.02 a, b, e, f, g, h, i, j, k, l, m	1.12 ± 0.06 a, d, e, f, g, h, i, k	1.10 ± 0.02 a, b, e, f, g, h, i, k	1.10 ± 0.04 a, b, d, e, f, g, h, i, j, k, l, m	1.11 ± 0.02 a, d, e, f, g, h, k, l, m
	30–40	1.11 ± 0.05 a, b, e, f, g, h, i, j, k, l, m	1.22 ± 0.08 c, e, f, g, k	1.13 ± 0.06 a, e, f, g, h, i, j, k	1.17 ± 0.05 a, c, d, e, f, g, h, k, l, m	1.22 ± 0.04 c, e, f, g, i, j, k, l
	40–50	1.25 ± 0.10 a, b, c, d, f, g, j, k, l, m	1.35 ± 0.09 c, d, f, i, j, k	1.30 ± 0.06 a, c, d, f, g, i, j, k	1.29 ± 0.06 a, c, d, e, k, l	1.32 ± 0.04 c, d, f, g, i, j, k, l
	50–60	1.38 ± 0.11 a, c, d, e, g, i, j, k, l, m	1.44 ± 0.06 b, c, d, e, h, i, j	1.45 ± 0.07 c, d, e, i, j	1.33 ± 0.08 a, c, d, e, k	1.43 ± 0.08 b, c, d, e, h, i, j, k, l
	60–70	1.44 ± 0.13 a, c, d, f, h, i, j, k, l	1.41 ± 0.07 b, c, d, h, i, j	1.49 ± 0.15 b, c, d, e, i, j	1.33 ± 0.11 a, c, d, k	1.44 ± 0.11 b, c, d, e, h, i, j, k, l
	70–80	1.33 ± 0.13 a, b, c, d, g, i, j, k, l, m	1.32 ± 0.08 c, f, g, j, k	1.42 ± 0.19 a, c, d, i, j	1.33 ± 0.12 a, c, d, k	1.30 ± 0.13 c, f, g, i, j, k, l
	80–90	1.20 ± 0.11 a, b, c, d, f, g, h, k, l, m	1.23 ± 0.08 c, f, g, j, k	1.27 ± 0.19 a, c, d, f, g, h, j, k	1.26 ± 0.15 a, c, k, l	1.11 ± 0.07 a, d, e, f, g, h, k, l, m
	>90	1.13 ± 0.09 a, b, e, f, g, h, k, l, m	1.17 ± 0.06 e, f, g, h, i, k	1.12 ± 0.06 a, e, f, g, h, i, k	1.23 ± 0.13 a, c, k	1.08 ± 0.03 a, d, e, f, g, h, k, l, m
Trunk extension	0–10	2.14 ± 0.56 b, c, d, e, f, g, h, i, j	1.81 ± 0.45 b, c, d, e, h, i, j	1.90 ± 0.30 b, c, d, e, i, j	2.32 ± 0.71 b, c, d, e, f, g, h, i, j	2.20 ± 0.34 a, b, c, d, e, f, g, h, i, j
	10–20	2.34 ± 1.18 b, c, d, e, f, g, h, i, j	1.78 ± 0.70	1.67 ± 0.41	2.09 ± 0.80 b, c, d, e, i, j	2.23 ± 0.63 b, c, d, e, f, g, h, i, j
	20–30	2.12 ± 1.21 c, d, e, f, h, i, j	1.77 ± 1.12	1.93 ± 0.82	2.13 ± 1.20 c, d	2.01 ± 0.61 c, i, j
	30–40	1.97 ± 1.70	1.63 ± 1.71	1.73 ± 0.43	1.76 ± 1.20	1.91 ± 1.25
	>40	1.47 ± 1.52	1.44 ± 1.88	1.40 ± 0.60	1.66 ± 1.71	1.19 ± 0.91

Note: a Statistical difference to 0°–10° (flexion), b statistical difference to 10°–20° (flexion), c statistical difference to 20°–30° (flexion), d statistical difference to 30°–40° (flexion), e statistical difference to 40°–50° (flexion), f statistical difference to 50°–60° (flexion), g statistical difference to 60°–70° (flexion), h statistical difference to 70°–80° (flexion), i statistical difference to 80°–90° (flexion), j statistical difference to >90° (flexion), k statistical difference to 0°–10° (extension), l statistical difference to 10°–20° (extension), m statistical difference to 20°–30° (extension).
